# Why Herd Size Matters – Mitigating the Effects of Livestock Crashes

**DOI:** 10.1371/journal.pone.0070161

**Published:** 2013-08-01

**Authors:** Marius Warg Næss, Bård-Jørgen Bårdsen

**Affiliations:** 1 Center for International Climate and Environmental Research – Oslo (CICERO), Fram Centre, Tromsø, Norway; 2 Norwegian Institute for Nature Research (NINA), Arctic Ecology Department, Fram Centre, Tromsø, Norway; Umea University, Sweden

## Abstract

Analysing the effect of pastoral risk management strategies provides insights into a system of subsistence that have persevered in marginal areas for hundreds to thousands of years and may shed light into the future of around 200 million households in the face of climate change. This study investigated the efficiency of herd accumulation as a buffer strategy by analysing changes in livestock holdings during an environmental crisis in the Saami reindeer husbandry in Norway. We found a positive relationship between: (1) pre- and post-collapse herd size; and (2) pre-collapse herd size and the number of animals lost during the collapse, indicating that herd accumulation is an effective but costly strategy. Policies that fail to incorporate the risk-beneficial aspect of herd accumulation will have a limited effect and may indeed fail entirely. In the context of climate change, official policies that incorporate pastoral risk management strategies may be the only solution for ensuring their continued existence.

## Introduction

More of the Earth’s land surface is used for grazing than for any other purpose [1]. Extensive pastoral production occurs in 25% of the global land area from the drylands of Africa and the Arabian Peninsula, to the highlands of Asia and Latin America and the Arctic parts of Fennoscandia and Russia [2]. Specifically, grazing land covers 77% of Australia, 61% of Africa, 49% of Asia and 18% of Europe [1]. It has been estimated that pastoralists produce 10% of the world’s meat, and supports some 200 million pastoral households who raise nearly 1 billion head of camel, cattle and smaller livestock [2]. The main livestock species kept by pastoralists are cattle, donkeys, goats and sheep, although they also keep, e.g., alpaca and llamas in the Andes, camels and horses in east-central Asia, the dromedary in Africa and West Asia, reindeer in northern Eurasia, and yak on the Tibetan Plateau [1]. Pastoralism is also economically important, especially in poor regions: compared to settled farmers in Africa, pastoralists produce 50–70% of all the milk, beef and mutton produced on the continent and while comprising only 1.5% of the total population of Iran, pastoralists keep 25% of the national herd [1]. From a global point of view, >1 billion people depend on livestock, and 70% of the 880 million rural poor living on less than USD 1.00 per day are at least partially dependent on livestock for their livelihoods [3]. Accordingly, Dong et al. [4] argue that pastoralism is important from a global point of view because of: (1) the human populations it supports; (2) the food and ecological services it provides; (3) the economic contributions it makes; and (4) the long-standing societies it helps to maintain.

### Environmental Hazards and Pastoralism

Environmental hazards, such as drought, floods and icing significantly affect livestock survival and reproduction. For example, in Africa mortality rates for cattle during drought have been estimated to be between 35–75% [5] and 10–25% [6], and 25% on average [7]. Small stock losses have been found to range between 1–35% (mean = 24.2%) for sheep and between 5–30% (mean = 16.6%) for goats [6]. Drought has also been found to increase the number of stockless households from 7 to 12% [7]. As for Mongolia, it has been reported that icing in 1993 resulted in the deaths of three-quarter of a million head of livestock where 110 households lost all animals, and 2 090 households lost >70% of their herds [8]. Between 1999 and 2002, 12 million livestock died in winter disasters, and many thousands of households lost their livelihoods [9]. In Inner Mongolia, about 30% of households have lost nearly all their livestock since 2001 due to continuous drought conditions [10]. On the Tibetan Plateau, six harsh winters with heavy snowfall from 1955 to 1990 resulted in 20–30% livestock losses [11]. Specifically, during the winter of 1996–1997 nomads on the western part of the plateau experienced losses of up to 70% and 25% of juvenile and adult goats respectively, and 20% of their lambs [11]. For Tibet in general, some townships lost up to 70% of their total livestock population, and by April 1998 it was estimated that the region had lost over 3 million head of livestock, which represents an estimated loss of USD 125 million [11]. In northern Norway, the reindeer husbandry utilizes winter pastures characterized by a cold but stable continental climate [12]. Nevertheless, mass starvation due to severe winter conditions, i.e. icing events, have been reported to dramatically reduce reindeer populations: In 1918 one reindeer population was, for example, reduced by a third [13], and adverse weather events, i.e. too much snow in late winter, also caused substantial reductions in 1958, 1962 and 1968 [14].

The effects of environmental hazards are especially important in the context of climate change because the frequency of extreme weather events are predicted to increase in the future (a trend that has already been observed empirically: e.g. [15], [16]), and thus represents a significant challenge for pastoralists [17]. It is therefore important to increase our understanding of both impacts of environmental hazards and the effects of strategies aimed at dampening them to enhance the ability of pastoralists to deal with the negative impacts of climate change.

### Predictions

Herd accumulation has been argued, as well as to some extent demonstrated empirically, to be an effective strategy for buffering environmental hazards for short periods of time because it seems that wealthier pastoral households weather calamities better than poorer ones [18]. Nevertheless, few studies have evaluated the long-term effect of herd accumulation (but see [19]) and a case has been made that the effectiveness of herd accumulation should be assessed by analysing changes in livestock holdings during crisis periods, i.e. when pastoralists experience a near collapse in livestock holdings [20].

Consequently, our study assess two predictions: First, while the relationship between pre-collapse herd size and losses due to the crisis should be positive, adding one animal to pre-collapse herd size should result in losing less than one animal during the crisis if herd accumulation is an effective strategy for countering the negative impacts of environmental hazards (this is in line with evidence from Africa, see [21]). Second, we also assess to what extent pre-collapse herd size predicts post-collapse herd size, i.e. the *per se* benefit that herders gain by adding animals to their herds [7], [20]. In short, we expect pre-collapse herd size to be a positive predictor of post-collapse herd size, if herd accumulation is an effective strategy for countering the negative impacts of environmental hazards.

## Materials and Methods

### Ethics Statement

The data utilized in this study were provided by the Norwegian Institute of Nature Research as part of the participation in the project ECOPAST (http://pastoralism-climate-change-policy.com/projects/). The standard of ethics pertaining to the data has been approved by the Norwegian Social Science Data Services in connection with the project ‘Beregning av produktivitet i reindrift’ (‘Calculation of productivity in the reindeer husbandry’).

### The Saami Reindeer Husbandry in Norway

Saami reindeer husbandry has been said to be the cornerstone of the Saami culture in northern Fennoscandia [22]. Although it is difficult to come up with accurate dating of the origin of reindeer husbanding as a pastoral economy, it developed at least 400 years ago [23] and probably evolved from a hunting culture based on wild reindeer. Traditionally, reindeer pastoralism was based on families, or households, which followed the herds year-round where the pastoral economy was based on reindeer products [24]. The reindeer husbandry has undergone major technological, economic and political changes; most notably the production system has changed from being subsistence based to a motorized and market-oriented industry [25]. During the late 1970s, the Norwegian Government became more directly engaged in the reindeer husbandry through subsidies and regulations. Reforms during the end of the 1970s and early 1980s aimed at increasing both production and co-management [26]. In 1976, negotiation between the Saami Reindeer Herders’ Association of Norway and the Norwegian Government resulted in the General Agreement for the Reindeer Industry (GARI). Importantly, this laid the foundation for annual negotiations pertaining to official subsidies and development: an arrangement that continues to this day [26]. The Reindeer Management Act (RMA) from 1978 focused on: (1) the establishment of formal institutions for access to the reindeer husbandry and pasture management; and (2) co-management. Berg [27] argued that the RMA of 1978 and the GARI of 1976 provided the foundation for a change into a corporative reindeer husbandry, i.e. not only production of meat for subsistence and sale but also for official subsidies. Accordingly, in many areas different support and compensatory arrangements have provided around half of the income [27]. The RMA from 2007 broadened the focus on co-management by giving the industry more self-determination, influence and responsibility for its actions [28].

At present, the Saami reindeer husbandry can be distinguished into three different levels of social organization. The *husbandry unit*, lately designated as ‘siida shares’, is the basic unit of the social organization and consists of a government license that entitles a person to manage a herd of reindeer within a delimited area. The *siida* is a cooperative unit composed of one or more reindeer management families organized on the basis of kinship joined together in social and labour communities for keeping control of herds of reindeer through herding. A *district* is a formal management unit with responsibility to provide the Norwegian Reindeer Husbandry Administration with information as well as ensuring that the reindeer husbandry is managed in accordance with governmental regulations [18], [29], [30], [31].

### Data Material – Collapse in the Reindeer Husbandry

**Figure 1:**
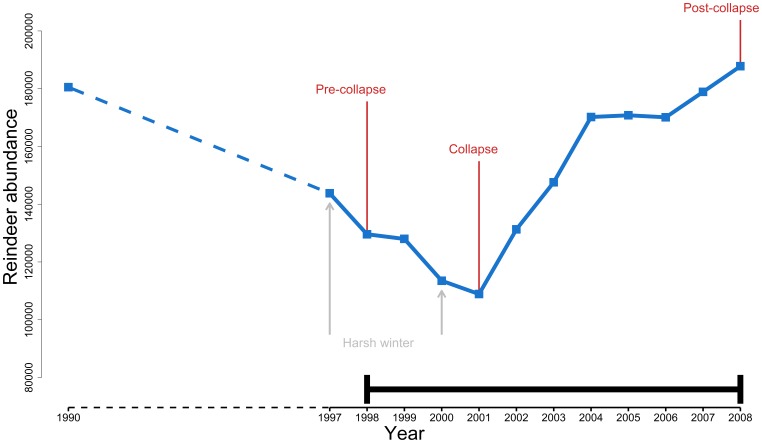
Temporal trend in reindeer abundance in Finnmark, Norway. Thick arrow indicates the period for which there exists official statistics pertaining to herd size for *individual husbandry units* (1998–2008), and thus restricts the period designations ‘pre-collapse’, ‘collapse’ and ‘post-collapse’ used in the analyses. Hatched line indicates missing data (i.e. abundance between 1990 and 1997). Abundance for 1990 from Tømmervik and Riseth [34], while abundance from 1997–2008 are per 31^st^ March for each year from Anonymous [65], [66], [67].

To evaluate the efficiency of herd accumulation as a risk reducing strategy the present study utilized data from the reindeer husbandry in Finnmark, Norway (see Text S1 for details). From an historical point of view, the number of reindeer in Finnmark has been characterized by considerable temporal variation: from the early 1900s, there was a decreasing trend that reached a minimum around the Second World War. Afterwards, while fluctuating there has been an upward trend that peaked in the early 1990s (from 90 000 animals in 1976 to 210 000 in 1988, [32]) and decreased until 2000/2001. More recently, reindeer abundance increased by ∼40% from 2002 to 2010 [33] and again reached a historical high-level [34]. After the peak in the early 1990s, governmental subsidies resulted in increased harvest rates and a subsequent decline in reindeer abundance [35]. This downward trend was further compounded by the “[…] catastrophic winters with heavy snowfalls in 1997 and 2000 […]” (Hausner et al. [14], p. 6), which resulted in a low point in reindeer abundance in 2001 (Fig. 1).

By designating the low point in 2001 (Fig. 1) as a ‘collapse’ in reindeer abundance (but see Text S2) it was possible to shed additional light on the effectiveness of herd accumulation as a risk management strategy by looking at the relationship between: (1) pre-collapse herd size and loss; and (2) pre-collapse and post-collapse herd size.

### Study Protocol and Statistical Analyses

As in previous studies, e.g. [36], this empirical study is based on governmental statistics compiled and published annually by the Norwegian Reindeer Husbandry Administration. This dataset contains data on herd size (total number of reindeer in the spring per husbandry unit), covering the period 1998–2008 with data from 20 reindeer husbandry summer districts. Data pertaining to herd size are based on counts made by herders that are regularly checked by the authorities (for more details pertaining to dataset and design, see [18], [29], [36]). The utilized dataset contains the following variables:


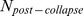
 (response).– A continuous (husbandry unit level) variable denoting the total herd size at the end of the period (i.e. 2008).


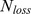
 (response).– A continuous (husbandry unit level) variable denoting the number of reindeer lost from the *pre-collapse* (1998) to the *collapse* (2001) year. The variable was created by subtracting herd size in 2001(collapse) from herd size in 1998 (pre-collapse).


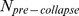
(predictor).– A continuous (husbandry unit level) variable denoting the total herd size at the beginning of the period (i.e. 1998).

To evaluate the efficacy of herd accumulation as a risk reducing strategy, we looked at the relationship between: (1) 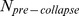 and 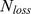; and (2) 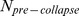 and 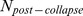. Statistical analyses and plotting of results were carried out in *R* [37]. All tests were two-tailed and the null-hypothesis was rejected at an *α*-level of 0.05, and we used Wald statistics to test if estimated parameters were significantly different from zero. Regular linear regression was used to investigate the overall relationships between pre-collapse and post-collapse herd size (grouping effects, e.g. possible differences between districts with respect to natural and/or social factors, were considered to be negligible, see Text S3 for details). Visual inspection of the data indicated problems related to the homoscedastic assumption [38]. We therefore fitted models with different variance structures in order to assess if violations of the homoscedastic assumption altered our conclusions [38]. As the conclusions from homoscedastic and heteroscedastic models were similar, we present the results from regular linear models in the main text (see Text S4 for the results based on heteroscedastic models).

## Results

**Figure 2:**
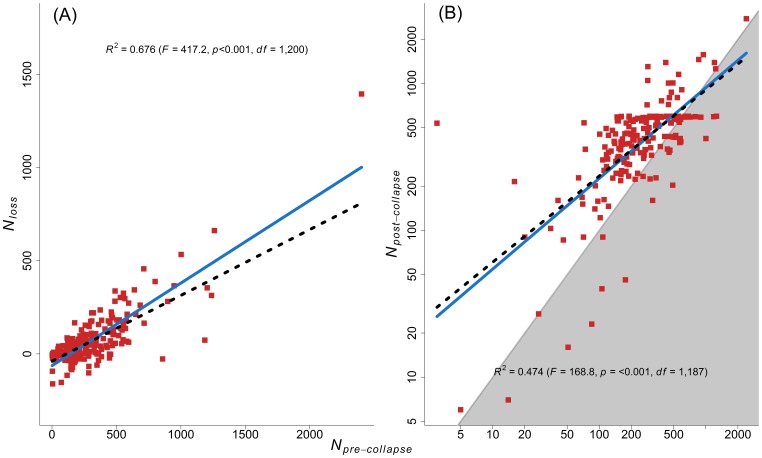
Showing the linear relationship between pre-collapse herd size (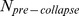) and number of animals lost from pre-collapse to collapse (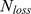) (A). Model parameters: Intercept = 75.24 [95% confidence intervals (CI): 63.42, 87.07] and slope (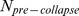) = 0.44 (95% CI: 0.40, 0.49). The positive relationship indicates that as herd size increases losses also increases: increasing herd size by one animal in 1998 increases the expected losses by 0.44 reindeer. Note that the model parameters are from fitting a model when centring 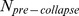 while the plot shows the relationship on the original scale. Hatched line show the relationship from a Generalized Least Square (GLS) model accounting for potential residual heterogeneity (see Text S4 for details). Showing the linear relationship (on log_e_-scale) between pre-collapse (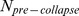) and post-collapse (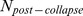) herd size (**B**). Points above the shaded area indicate herd increase over the period, while a point on the 45-degree line means that pre- and post-collapse herd size was equal, and points in the shaded region indicate a decrease. The cloud of points above the 45-degree shaded area reflects the overall increase in reindeer abundance for the study area (Fig. 1). Model parameters: Intercept = 2.58 (95% CI: 2.06, 3.09) and slope (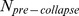) = 0.62 (95% CI: 0.52, 0.71). The positive relationship indicates that as pre-collapse herd size increases so does post-collapse herd size: a 1% increase in 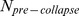 herd size predicts a 0.62% increase in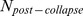 herd size. Hatched line show the relationship from a GLS model accounting for potential residual heterogeneity (see Text S4 for details).

Pre-collapse herd size had a positive effect on the number of animals lost [effect of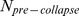: 0.44], which means that husbandry units with more animals in 1998 tended to lose more animals from 1998 to 2001 (Fig. 2A). More specifically, if a herder increased his/her herd by one animal in 1998 his/her losses were expected to increase by 0.44 animals. This indicates that while herd accumulation is effective, it is a costly form of insurance against environmental variability. The heteroscedastic models revealed the same relationship although the estimated effect size was reduced (see Text S4 for details). For a similar analysis pertaining to reported losses, see Text S5.

Pre-collapse herd size had a positive effect on post-collapse herd size [effect of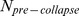: 0.62], i.e. husbandry units that had more animals in 1998 also tended to have more animals in 2008 (Fig. 2B). While the relationship is not perfect, it provides the underlying rationale for herd accumulation: having a large herd prior to a collapse ensures a large herd after the collapse (a 1% increase in pre-collapse herd size predicts a 0.62% increase in post-collapse herd size, as both variables were log_e_-transformed). Herd accumulation thus seems to be an effective strategy for countering the negative impacts of environmental hazards. The heteroscedastic models revealed the same relationship, but again the effect size was reduced (see Text S4 for details).

## Discussion

The positive relationship between pre- and post-collapse herd size indicates that herd accumulation is a rational response to environment-induced catastrophes, and provides the rationale for why both reindeer herders [29], [31] and pastoralists in general [39] invest labour to increase herd size. Nevertheless, herd accumulation may also stem from cultural values: e.g. prestige or status [40], [41], [42]; conspicuous display [43]; and provision of bridewealth [44]. However, the fact that pre- and post-collapse herd size was positively correlated clearly shows the economic rationale and whether herd accumulation also results from cultural values is somewhat irrelevant [20]. This point of view is also shared by the herders in Finnmark since 51% of the herders ‘agree’ or ‘strongly agree’ that herds size is an important risk reducing strategy, while only 26% ‘agree’ or ‘strongly agree’ that herd size is important for social status [45].

A previous study found that herd size one year was positively correlated with herd size the next year [18]. Although the previous study was based on a shorter time series, the present study complements the former in showing the underlying rationale for herd accumulation: (1) a large herd one year result in a larger herd the next year [18]; and (2) a large pre-collapse herd also result in a larger post-collapse herd (this study). In short, since herders with large herds also have comparable larger herds from one year to the next and during crisis periods, herd accumulation maximizes long-term survival for pastoralists (see also [46]). Nevertheless, the positive relationship between pre-collapse herd size and loss indicates that herd accumulation is a costly form of insurance against environmental variability (see [21] for a similar result for Africa; and see [47] p. 36-8 for a discussion pertaining to ‘loss’ and the effectiveness of herd accumulation).

Herd accumulation may, however, not be the best available risk reducing strategy because of negative density dependence. For the reindeer husbandry it has been demonstrated that reindeer density and climatic conditions have negative effects on individual body mass [12], [31], [48], [49], [50] and, consequently, also survival [49], [51]. Increased reindeer abundance also increases herders’ vulnerability to unfavourable climate as the negative impacts of adverse climatic events increases with increasing reindeer density [12], [49], [52]. From a risk perspective, the best long-term strategy may thus be to invest in livestock body mass and not herd size. Næss et al. [29] hypothesised that one reason for why this strategy is not utilised by herders is because of competition for access to common winter pastures since access is to a large degree determined by herd size. Herd accumulation, through e.g. cooperative labour investment [29], may thus be a viable strategy for gaining access to winter pastures. More to the point, competition for access to winter pastures may explain why herd accumulation is the dominant and only viable risk reducing strategy in Finnmark. Herd accumulation may thus be taken to support a Tragedy of the Commons (ToC) [53] situation in Finnmark. ToC is a prisoners’ dilemma where everybody is better off by coordinating strategies, e.g. by restricting the number of animals on the pastures, but the dominant solution is to always maximize herd size because the cost of high reindeer density is shared between all herders while the benefit of adding additional animals is attached to individual units [18]. Nevertheless, evidence supports both a *presence*, e.g. the aforementioned negative density dependence, and an *absence*, e.g. a positive relationship between slaughter undertaken by neighbouring herders and own slaughter [30], of ToC in Finnmark.

### Future Prospects and Management Implications

Scenarios for future climate change generally predict an increasing average, variance and even a changed distribution of important climatic variables like precipitation and temperature [15], [54]. Climate change will most likely result in more frequent extreme weather events and there are indications that extreme events, such as heat waves and precipitation extremes, will increase and already have done so [15], [16]. Nevertheless, there is limited information available pertaining to how these changes are going to affect the many pastoral cultures of the world. In Africa, climate change is predicted to increase the variability and frequency of rainfall at the same time as the proportion of arid and semiarid lands is likely to increase by 5–8% by 2080 [55]. Furthermore, in the Greater Horn of Africa droughts have now become the norm rather than the exception [56]. While previously pastoralists experienced one major long-term drought every decade coupled with minor occurrences every 3–4 years, droughts now occur annually [56]. As for the Arctic and Sub-Arctic, scenarios generated by most global climate models predict that the climate is likely to become increasingly unstable during the next half century with concomitant increases in the frequency of extreme weather conditions [57]. For Mongolia, regional climate predictions anticipate an increase in areas affected by droughts and in the frequency of extreme events [58]. Importantly, the frequency of droughts has almost doubled during the last 60 years and the worst droughts on record (covering over 50–70% of the country) have occurred during the last decade [58].

Considering the negative impact that environmental hazards have on livestock survival and reproduction, climate change thus represents a significant threat for the future of pastoral societies on a global scale. Nevertheless, it has been argued that by reinforcing the traditional strategies pastoralists have developed to deal with climate variability, in addition to introducing newer techniques, the economic, social, and cultural well-being of pastoral societies can be supported in the face of climate change [59]. Moreover, a case has been made that pastoralists are in a unique position to tackle climate change due to extensive experience managing environmental variability in marginal areas [2] and it has been argued that the ability to withstand environmental shocks is a *defining* feature of pastoralism [60].

Nevertheless, traditional pastoral risk management strategies, such as herd accumulation, may be insufficient for dealing with climate change [56]. While herd accumulation seems to be an efficient strategy, it is predicated on periods of recuperation when herd growth is possible. In fact, a delay in restocking after environment-induced losses is one of the main problems of pastoral production [61]. Herd accumulation can thus be expected to work less efficiently, if at all, when the frequency of extreme events increases.

As for Finnmark, it could be argued that the number of reindeer in Finnmark is unsustainable high, witnessed by the presence of negative density dependence (see above). Consequently, the Norwegian Government is aiming to reduce the number of reindeer so as to achieve a sustainable balance between pasture resources and number of reindeer [28]. The primary tool utilized are several subsidies that aims to increase slaughter and thus reduce herd size [62], [63]. Considering the risk-beneficial aspects of having a large herd, this may, however be viewed as a short-term solution that, if followed, decreases long term viability by reducing the insurance potential of large herds. More to the point, official management strategies that fails to incorporate the risk-beneficial aspect of herd accumulation will have a limited effect and may indeed fail entirely [18], [36]. Furthermore, production subsidies alone may not properly account for the decision problem facing herders: how to secure a reliable income while at the same time maximizing long-term survival. This decision problem is even more dire in the face of climate change: if herd accumulation works less efficiently when the frequency of extreme events increases, governmental support that incorporates (or preferably increase the effect of) pastoral risk management strategies may be the only solution for ensuring the continued existence of pastoralism. In the end, it may be official policies that disregard the inherent risk reducing logic of pastoral strategies rather than climate change *per se* that represents the greatest challenge for pastoral adaptability [17], [64].

## Supporting Information

Text S1 **Study design and the reindeer husbandry.** This text provides a more detailed description of the study design as well as the reindeer husbandry in Norway.
(PDF)

Text S2 **Is there really a ‘collapse’ in reindeer abundance?** This text investigates temporal trends in low points of reindeer abundance from 1845–2000.
(PDF)

Text S3 **Possible grouping effects.** This text investigates possible grouping effects, e.g. differences between summer districts with respect to natural and/or social factors.
(PDF)

Text S4 **Finding the correct variance structure.** This text explores models with different variance structures in order to assess if violations of the homoscedastic assumption altered the conclusions presented in the main text.
(PDF)

Text S5 **Reported loss.** This text replicates the analysis pertaining to loss presented in the main text but where ‘reported loss’ is used as a response.
(PDF)
