# Supplementary material for: Why Herd Size Matters – Mitigating the Effects of Livestock Crashes
Source: PLoS One. 2013 Aug 1;8(8):e70161. doi: 10.1371/journal.pone.0070161 (PMC3731343; doi:10.1371/journal.pone.0070161)
Supplement: Text S1 — Study design and the reindeer husbandry. This text provides a more detailed description of the study design as well as the reindeer husbandry in Norway. (PDF) [file pone.0070161.s001.pdf]

Text S1 – STUDY DESIGN AND THE REINDEER HUSBANDRY

STUDY DESIGN

The empirical analyses presented here are based on data from the Saami reindeer husbandry in Norway. Different reindeer summer pasture areas (summer districts) represent heterogeneous units that differ in both climate and herding strategies [1]. Previous studies have used differences in density as a basis of a paired-block design between neighbouring areas with low and high density. This quasi-experimental design, consisting of 10 pairs and 20 districts from Finnmark, northern Norway, have been adopted to separate the effects of reindeer density from other environmental factors (cf. [2,3,4] and references therein for a description of details and the rationale behind the study design).

THE SAAMI REINDEER HUSBANDRY IN NORWAY – A BRIEF INTRODUCTION

Historical origins

Saami reindeer husbandry has been said to be the cornerstone of the Saami culture in northern Fennoscandia [5]. Although it is difficult to come up with accurate dating of the origin of reindeer husbanding as a pastoral economy, it developed at least 400 years ago ([6,7], but see e.g. [5,8,9,10] for other estimates) and probably evolved from a hunting culture based on wild reindeer. During glacial time in Fennoscandia, the presence of wild reindeer was most likely an important reason for stone-age settlement of hunting people [11]. According to Riseth [11] in older times the Saami adaptation was semi-nomadic, consisting of a limited number of households that migrated between different seasonal locations, and for which the hunting of wild reindeer was the most important economic activity. These bands were furthermore organized according to the term siida, who described both the territory used and the band: "The hunting siida can be defined as *an organization of households, which utilize common-pool-resources for hunting, trapping, and fishing in a geographic area and which claim exclusive rights to these resources*" ([11], p. 120, italics in original). Furthermore, from the 17th century, siida borders delineated property rights that were legally recognized by nation-state rulers [11]. By the end of the 16th and during the 17th century the Saami hunters used tame reindeer as draft animals and as decoys during hunts [11]. Increased hunting pressure on wild reindeer, coupled with the already use of tame animals, provided, according to Riseth [11], the basis for the transformation of the hunting culture to full nomadic reindeer pastoralism as tame reindeer provided a much more stable source of livelihood than the decreasing population of wild reindeer [10,12,13].

Riseth [11] has summarized the changes from a hunting culture to nomadic reindeer pastoralism as follows: (1) the siida areas became larger to incorporate the longer migration necessary for reindeer pastoralism; (2) a reduction of the number of households per siida; (3) the household became a more important economic unit; and (4) labour cooperation combined with a privatization of production. Reindeer pastoralism was traditionally based on families, or households that followed their herds year-round where the pastoral economy was primarily tied to reindeer products [12]. Central to the reindeer husbandry is the two levels of social organization: (1) *baiki* (household); and (2) *siida* [8,11,14,15]. The household consisted of a family group comprising a nuclear family but may have also included servants or old people e.g. the parents of the wife or husband [11]. In general terms, members of the household owned their own animals privately and made decisions in relation to slaughter and reproduction on their own, although under the supervision of the household leader [6]. New households were usually formed

upon marriage, i.e. reindeer has traditionally been individually owned and inherited by both women and men [7], and upon marriage both the groom and bride brought their own reindeer, which they have accumulated since birth, into the new household [16].

The *siida* can be defined as a cooperative unit composed of one or more reindeer management families, usually organized on the basis of kinship, which joined in a social and labour community for keeping control over a herd of reindeer through herding [15]. The siida was (and still is) both a social and a working community normally consisting of several households. Moreover, the siida was a flexible system of cooperation, i.e. siida membership could change over time so as to optimise the relationship between herds and personnel [7,17]. As the siida can be defined as a unit composed of one or more reindeer management families, Riseth and Vatn [6] argues that the siida carried with it potential sources of conflict related to differences between individual and collective interests. Consequently, the siida was traditionally regulated by a number of principles: (1) all herd owners were their own masters; (2) solidarity between siida partners, which sometimes could be stronger than sibling solidarity; (3) a network of mutual obligations; (4) unanimity in relation to collective decisions and (5) a spiritual land-man relationship. According to Riseth and Vatn [6] inter-siida conflicts where resolved through the use of 3-5.

Central concepts in reindeer husbandry are that of *herding* and *husbandry*: Paine [18] defines herding as the relationship between herd/pasture in connection to the welfare of the animals in the terrain, which was the responsibility of the siida group [14]. Husbandry on the other hand, related to the reindeer herd as a resource of its owners [18]. Husbandry for Paine ([18], p., 79) is "[…] the efforts of the owners in connection with the growth of capital and the formation of profit". Decisions in connection to husbandry were the responsibility of the household [14].

The reindeer husbandry in transition

The newer history of Saami reindeer husbandry can be summarized as being influenced by an increased meat and market adaptation coupled with an increased sedentarisation [19]. According to Riseth and Vatn [6] after the Second World War the life of the Saami reindeer herders changed as herding changed to become more an occupation than a way of life. Moreover, during the 1950s access to markets increased and from the middle of the 1960s the reindeer husbandry underwent major technological changes with the introduction of snowmobiles and later all-terrain vehicles (ATVs). During the late 1970s the Norwegian Government became more and more directly engaged in the reindeer husbandry through subsidies and regulations. Reforms during the end of the 1970s and early 1980s had as one of its main aims to increase both production and co-management [6].

Herd sizes in Finnmark were at their lowest after the end of the Second World War, but were normalised by the end of the 1950s. At the beginning of the 1950s the Norwegian Government tried to help reindeer herders to increase herds to a pre-war level by supplying capital for buying animals for breeding and by erecting fences between summer districts [6]. The Norwegian Government also started to build slaughterhouses throughout the 1950s something which gave herders good opportunities for slaughtering during the 1950s and 1960s. According to Riseth and Vatn [6] and Riseth [19] one of the main underlying ideas for increased governmental involvement in the reindeer husbandry was to rationalise the industry. During this period the reindeer husbandry underwent major technological, economic and political changes [11,20], which had as one of its consequence that, in Finnmark, one observed a doubling of

number of reindeer from around 1970 to 1990 [6], while the number of husbandry units doubled from 1950 to 1990 [6].

In Finnmark, the snowmobile was introduced in the middle of 1960 and was adopted by almost all herders within a relatively short time. Moreover, where it was possible, i.e. in areas with not too steep terrain, ATVs were being used during summer. Generally speaking, during this period the reindeer husbandry experienced a high degree of technological modernization within the limits set by economy and geography [6]. This was a change that, according to Riseth and Vatn [6], was welcomed by the reindeer herders. During this period cars and trucks were used more and more for transporting both reindeer and people. For example, trucks could be used to move the weakest animals or the whole herd during spring migration if necessary [6]. Moreover, both helicopters and military transport boats were used in the reindeer husbandry. More and more fences were also raised as well as a general modernisation of the corral system [6]. In sum, during this period the production system started to change from being subsistence based to a motorised and market oriented industry [6,11,20]. The technological change, illustrated by the increased dependence of e.g. snowmobiles, were financed by slaughtering, which according to Riseth and Vatn [19] and Nilsen and Mosli [14] provides a partial explanation for why the number of reindeer experienced a temporary decrease prior to and around 1970.

During the 1970s most of the reindeer herders had moved into modern houses in central areas in Finnmark. This was due to official policies such as housing programmes (1958 and 1969) that made modern houses affordable for herders. Moreover, nine years compulsory school made it impossible for families to live close to their herd [6]. Furthermore, the public sector grew considerably from the end of the 1960s and new ways of making money became more and more available, especially for women. All these factors made it possible for reindeer herders to attain a "modern" standard of living [6,14]. Furthermore, at the end of the 1970s a new step in governmental involvement in the reindeer husbandry was initiated. The Saami Reindeer Herders' Association of Norway (NRL)[1] lobbied for recognition and support for viewing the reindeer husbandry as an industry [6]. In short, they achieved two important goals: in 1976 the General Agreement for the Reindeer Industry was negotiated between NRL and the Norwegian Government. This agreement, approved by the Norwegian Parliament, achieved that reindeer pasture areas should be protected from encroachment from other industries and secured both welfare and income for Saami reindeer herders [6]. Far more important, however, is the fact that this agreement laid the foundation for annual agreements pertaining to official subsidies and development that continues to this day ([6], see also [22]). Riseth and Vatn [6] has argued that the subsidies had an unintended consequence in Finnmark: the increased income generated from e.g. subsidies was partly converted to larger herd sizes (see also [2]).

In 1978 a new Reindeer Management Act was adopted, focusing on: (1) the establishment of formal institutions for access to the reindeer husbandry and pasture management; and (2) co-management. While the establishment of formal institutions was based on the rationalisation and efficiency paradigm, co-management was based on herder representation in the administration of the reindeer husbandry, both regionally and nationally [6]. Locally, democratically elected districts boards were established for each reindeer district [22]. The intention was to establish a framework for governance that should limit the growth of both husbandry units and herds as well as making sure that reindeer herders and their representatives should be accountable for their decisions [6,22]. The number of husbandry units was controlled by the use of a quota system [23]. The Reindeer Management Act placed weight on active management for members of the

---

[1] The national interest organisation of the reindeer husbandry established in 1948 [21].

husbandry unit as well as restricted the possibility of keeping reindeer in other husbandry units [6]. Furthermore, the act also established a structure for governance where the boards were given authority in relation to e.g. herd quotas on both district and husbandry unit level [6]. According to Riseth and Vatn [6] several changes followed. First, the status of the husbandry unit seems to have been strengthened at the expense of the siida system (see also [14]). One of the reasons for this is that governmental subsidies targeted the husbandry unit. Second, at the same time husbandry units got increased opportunities for generating income from non-pastoral activities, i.e. increased sedentarisation lead to a closer link with the overall society which again increased income generating opportunities [6]. Berg [21] has argued that the Reindeer Management Act of 1978 and the General Agreement for the Reindeer Industry of 1976 lay the foundation for a change into a *corporative* reindeer husbandry, i.e. not only production of meat for subsistence and sale but also for official subsidies. Accordingly, in many districts it has been common that half of the income has been generated by different support and compensatory arrangements [21].

The focus on co-management has been broadened in the Reindeer Management Act of 2007. The committee in charge of proposing the 2007 Reindeer Management Act wanted "[…] more power-sharing between the government and industry and more influence on the part of reindeer owners […]" and that the "[…] industry should have self-determination and influence but also more responsibilities for its actions […]" ([22], p. 66). To accommodate increased co-management the traditional siida system has been recognised as an important managerial unit and where different siidas should elect boards that is to work as a contact point between the siida and the district boards [22,24]. In short, it is assumed that "[…] a well-arranged management system at the local level will lead to better social relations, increased trust and better co-operation among the reindeer owners […]" ([22], p., 68).

In sum, the reindeer husbandry have undergone substantial changes, the most important being: (1) more extensive management (decrease in contact between man and animals; see [25] for a discussion concerning the erosion of herding skills); (2) the subsistence economy was replaced by a money-based economy; (3) sedentarisation; (4) reindeer husbandry changed from a way of life to an occupation; (5) an increase in the use of modern technology; and (6) increased dependence on governmental support [6,7,10,11,17,21,23]. Also, Bjørklund [23] argues that while in traditional reindeer husbandry the principal factors of production were under the control by traditional institutions, during 1960-90 the Norwegian Government assumed the control by e.g. setting a limit on the number of animals, and by controlling the recruitment of reindeer herders by the use of a quota system.

Organisation and administration

According to Ulvevadet [22], management of the reindeer husbandry consist of a complex co-management system with participants from the bottom to the top. Moreover, "[…] there are three organizational systems with vertical and horizontal interaction among all its organizational parts" ([22], p. 55). First is the administrative system that goes from the Parliament to the Ministry of Agriculture and Food and further to the Reindeer Husbandry Administration with its six different reindeer husbandry areas at the regional level. Accordingly the six different reindeer husbandry areas "[…] provide reindeer owners with assistance and advice […] ([22], p. 65). Another part of the system consist of the corporative system that goes from NRL to six regional associations located within the six different reindeer husbandry areas [22]. As previously mentioned NRL negotiates with the government concerning annual agreements pertaining to official subsidies and development of the reindeer husbandry [6,22]. Finally, there is an extensive co-management

system that consists of different boards within the: (1) Reindeer Husbandry Administration; (2) the six different reindeer husbandry areas; (3) reindeer districts; and (4) siidas. “Reindeer owners are the sole members of the district boards. Members of the area boards are appointed by the Sami Parliament and the County Council […], while members of the Reindeer Husbandry Board are appointed by MAF [Ministry of Agriculture and Food] and the Sami Parliament” ([22], p. 55). Members of the siida board are elected from the reindeer herders within the siida.

As for social organization, at present Saami reindeer husbandry operates at three different levels of social organization: (1) husbandry unit; (2) siida; and (3) district. The husbandry unit is the basic unit of the social organization, and is licensed by the government to manage a herd of reindeer within a delimited area [26]. The husbandry unit is similar to the household as defined by Dahl [27], but as the herd can also contain reindeer belonging to family members of the husbandry unit’s manager, it resembles an extended family unit. The siida is a cooperative unit composed of one or more reindeer management families, and is part of the traditional reindeer husbandry system[2] [3]. The siida is usually organized on the basis of kinship joined together in social and labour communities for keeping control of herds of reindeer through herding [3]. Saami kinship system is extensive and includes terms for consanguinal and affinal relationships [15]. Traditionally, Saami kinship system was bilateral, i.e. kinship defined through both the male and female lines [28]. Sibling solidarity, however, could be extended to include cousins and other affinal relatives of the same generation [29].

Saami reindeer husbandry districts are formal management units with responsibility to provide the Norwegian reindeer husbandry administration with information. The district is also responsible for ensuring that reindeer husbandry is managed in accordance with government regulations [30]. As such the district might be better described as the lowest level of government management of the reindeer industry rather than a level of social organization [22] even though members of reindeer districts have to cooperate in, e.g. maintaining fences or fulfilling governmental quotas on the maximum number of reindeer per district [3,31].

Pasture use

Important prerequisites for the reindeer husbandry have arguably been land, herd and personnel [6,7,32]. Pastures are, however, seasonal and have specific physical and geographical locations than necessitates migration between them. For reindeer, the most important diet during winter is ground lichens which are commonly distributed in relatively dry continental areas [6]. During summer, reindeer subsist mostly on herbs and grasses which are most commonly distributed in nutritious mountain areas. Spring and autumn pastures usually consist of both this types of resources [6]. Riseth and Vatn [6] argues that as lichens are a stock resource that experience optimal growth during relatively low grazing pressure, the capacity of winter grazing areas determines to a large degree herd size. In contrast, the capacity of the green summer pastures determines the production potential [6,33]. In short then, herding of reindeer is based on following the natural migration patterns of reindeer. In Finnmark, the summer pastures are localized in the northeast close to the coast and fjords while winter pastures are on the continental inland plateau. Summer pastures are moreover geographically delineated on the basis of *district* borders, i.e. the aforementioned formal management units. Summer pastures are shared between several husbandry units. The combined spring and autumn pastures are localized between the

[2] This level of social organization is *formally* recognized by the Norwegian Government in the new Reindeer Management Act as what has previously been designated as *husbandry unit* will change to *siida share* [22,24].

summer and winter pastures (see Fig. 1 in [3] for a visualization). Spring, autumn and winter pastures consist of large common grazing areas that in some periods include five to six dozen winter siidas [6]. In short, the distribution of pastures requires that Saami reindeer herders living in Finnmark migrate with their reindeer out to costal summer pastures during spring time from winter pastures in the interior with distances often being several hundred kilometres [34].

REFERENCES CITED

1. Bårdsen B-J, Tveraa T (2012) Density dependence vs. density independence – linking reproductive allocation to population abundance and vegetation greenness. Journal of Animal Ecology 81: 364-376.
2. Næss MW, Bårdsen B-J (2010) Environmental Stochasticity and Long-Term Livestock Viability-Herd-Accumulation as a Risk Reducing Strategy. Human Ecology 38: 3-17.
3. Næss MW, Bårdsen B-J, Fauchald P, Tveraa T (2010) Cooperative pastoral production - the importance of kinship. Evolution and Human Behavior 31: 246-258.
4. Næss MW, Bårdsen B-J, Pedersen E, Tveraa T (2011) Pastoral herding strategies and governmental management objectives: predation compensation as a risk buffering strategy in the Saami reindeer husbandry. Human Ecology 39: 489-508.
5. Bostedt G (2001) Reindeer husbandry, the Swedish market for reindeer meat, and the Chernobyl effects. Agricultural Economics 26: 217-226.
6. Riseth JÅ, Vatn A (2009) Modernization and Pasture Degradation: A Comparative Study of Two Sàmi Reindeer Pasture Regions in Norway. Land Economics 85: 87-106.
7. Paine R. (1994). *Herds of the Tundra: a portrait of Saami reindeer pastoralism*, Washington London.
8. Bjørklund I (1990) Sami Reindeer Pastoralism as an Indigenous Resource Management System in Northern Norway: A Contribution to the Common Property Debate. Development and Change 21: 75-86.
9. Hansen LI, Olsen B. (2004). *Samenes historie*, Oslo: Cappelen akademisk.
10. Bergstrøm C (2005) Claiming reindeer in Norway: towards a theory of the dynamics of property regime formation and change. Ås: Norwegian University of Life Sciences. 293 p.
11. Riseth JÅ (2000) Sámi reindeer management under technological change 1960-1990: implications for common-pool resource use under various natural and institutional conditions: a comparative analysis of regional development paths in West Finnmark, North Trøndelag, and South Trøndelag/Hedmark, Norway [Doctor scientarium]. Ås: Norges landbrukshøgskole. 2 b. (239 s.) p.
12. Vorren Ø. (1978). *Bosetning og ressursutnytting under veidekulturen og dens differensiering*, in NOU 18A, Finnmarksvidda-natur-kultur. Oslo: Universitetsforlaget. pp. 145-62.
13. Holand Ø. (2003). *Reindrift - samisk næring i brytning mellom tradisjon og produksjon*, Oslo.
14. Nilsen R, Mosli JH. (1994). *Inn fra vidda: hushold og økonomisk tilpasning i reindrifta i Guovdageaidnu 1960-1993*, Guovdageaidnu: BAJOS Utviklingsselskap AS/NORUT Samfunnsforskning AS, Tromsø.
15. Pehrson RN. (1964). *The bilateral network of social relations in Könkämä Lapp district*, Oslo: Universitetsforlaget.
16. Paine R. (2009). *Camps of the tundra: politics through reindeer among Saami pastoralists*, Oslo: Instituttet for sammenlignende kulturforskning.
17. Bjørklund I (2004) Saami Pastoral Society in Northern Norway: The National Integration of an Indigenous Management System. In: Anderson DG, Nuttall M, editors. Cultivating

Arctic Landscapes: Knowing and Managing Animals in the Circumpolar North. New York: Berghahn. pp. 124-135.
18. Paine R (1964) Herding and husbandry: two basic distinctions in the analysis of reindeer management Folk 6: 83-88.
19. Riseth JÅ (2006) Sàmi reindeer herd managers: why do they stay in a low-profit business? British Food Journal 108: 541-559.
20. Riseth JÅ (2003) Sami Reindeer Management in Norway: Modernization Challenges and Conflicting Strategies. Reflections Upon the Co-management Alternative. In: Jentoft S, Minde H, Nilsen R, editors. Indigenous Peoples: Resource Management and Global Rights. Delft, Netherlands: Eburon Academic Publishers. pp. 229-247.
21. Berg BÅ (2008) Utviklingen av reindriften i nordre Nordland 1750-2000. In: Evjen B, Hansen LI, editors. Nordlands kulturelle mangfold: etniske relasjoner i historisk perspektiv. Oslo: Pax. pp. 151-191.
22. Ulvevadet B (2008) Management of reindeer husbandry in Norway - power-sharing and participation. Rangifer 28: 53-78.
23. Bjørklund I. (1999). *Norsk ressursforvaltning og samiske rettighetsforhold: om statlig styring, allmenningens tragedie og lokale sedvaner i Sápmi*, Oslo: Ad Notam Gyldendal.
24. Anonymous (2007) Lov om reindrift av 15. juni. 2007 nr 40.
25. Beach H (2000) Pastoralism Politics in Sweden: Protecting the Environment and Designing the Herder. In: Hornborg A, Pálsson G, editors. Negotiating Nature: Culture, Power, and Environmental Argument. Lund: University Press.
26. Ulvevadet B, Klokov K, editors (2004) Family-based reindeer herding and hunting economies, and the status and management of wild reindeer/caribou populations. Tromsø: University of Tromsø, Centre for Saami Studies 170 s. p.
27. Dahl G. (1979). *Suffering grass: subsistence and society of Waso Borana*, Stockholm: Department of social anthropology University of Stockholm.
28. Gjessing G (1975) Socio-Archeology. Current anthropology 16: 323-332.
29. Bergman I, Liedgren L, Östlund L, Zackrisson O (2008) Kinship and Settlements: Sami Residence Patterns in the Fennoscandian Alpine Areas around A.D. 1000. Arctic Anthropology 45: 97-110.
30. Bull KS. (1997). *Studier i reindriftsrett*, Oslo: Tano Aschehoug.
31. Næss MW, Fauchald P, Tveraa T (2009) Scale Dependency and the "Marginal" Value of Labor. Human Ecology 37: 193-211.
32. Paine R (1972) The Herd Management of Lapp Reindeer Pastoralists. In: Irons W, Dyson-Hudson N, editors. Perspectives on Nomadism. Leiden: E.J. Brill. pp. 76-87.
33. Tveraa T, Fauchald P, Yoccoz NG, Ims RA, Aanes R, et al. (2007) What regulate and limit reindeer populations in Norway? Oikos 116: 706-715.
34. Paine R (2004) Saami Reindeer Pastoralism: Quo Vadis? Ethnos 69: 23-42.
