# Supplementary material for: Why Herd Size Matters – Mitigating the Effects of Livestock Crashes
Source: PLoS One. 2013 Aug 1;8(8):e70161. doi: 10.1371/journal.pone.0070161 (PMC3731343; doi:10.1371/journal.pone.0070161)
Supplement: Text S2 — Is there really a ‘collapse’ in reindeer abundance? This text investigates temporal trends in low points of reindeer abundance from 1845–2000. (PDF) [file pone.0070161.s002.pdf]

Text S2 – IS THERE REALLY A 'COLLAPSE' IN REINDEER ABUNDANCE?

INTRODUCTION

As stated in the main text, the number of reindeer in Finnmark, Norway has been characterized by considerable temporal variation: from the early 1900s there was a decreasing trend that reached a minimum around the Second World War. Afterwards, there has been an upward trend that peaked in the early 1990s, decreased until 2000/2001 and has experienced an increase in recent years (in 2010 the number of reindeer reached the historical level of 1990 [1]). Similarly, historical data indicates that there were fluctuations in reindeer abundance in Finnmark between 1835 and the early 1900s [1]. More to the point, while this period is characterized by several low points in abundance, it seems that there is a positive temporal trend for the low points, i.e. the low points are characterized by increasing abundance (Fig. S2.1). Nevertheless, this positive trend was not statistically significant (Fig. S2.1) and the period designated as a 'collapse' in the main text represent a low point for which there exists data pertaining to individual husbandry units (Fig. 1 in the main text).

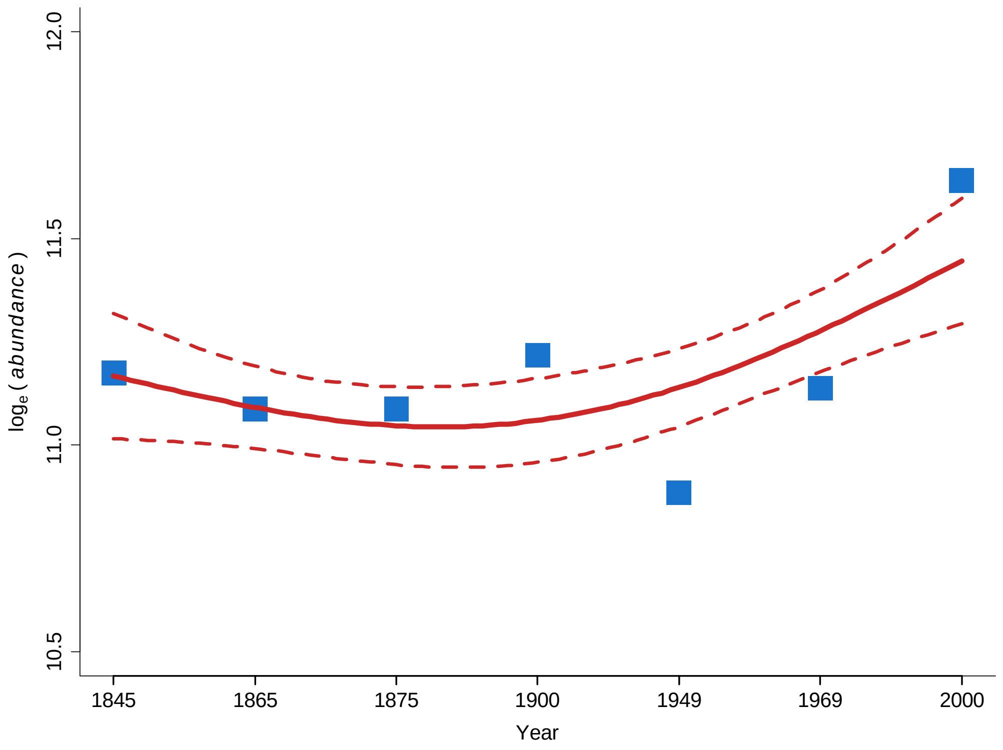

Fig. S2.1 The temporal trend in low points of reindeer abundance in Finnmark (low points and data from Fig. 4 and table 2 in [1]). Predictions from a Generalized Additive Model (GAM), with a Gaussian family and the identity link-function, was used to visualize the relationship between year and abundance on $\log_e$-scale [GAM results: Intercept = 11.175 (SE = 0.0704, $p < 0.001$); s(year) = 1.713 ($p = 0.188$, $k = 3$)]). Deviance explained by the model: 53.4%. Please note that the original data was presented at ~10 years intervals and as such may hide important trends, e.g. there may be low points within the periods not covered by the data.

REFERENCES CITED

1. Tømmervik H & Riseth JÅ (2011) Historiske tamreintall i Norge fra 1800-tallet fram til i dag. in *NINA Rapport* (NINA). Tromsø: NINA.
