# Supplementary material for: Why Herd Size Matters – Mitigating the Effects of Livestock Crashes
Source: PLoS One. 2013 Aug 1;8(8):e70161. doi: 10.1371/journal.pone.0070161 (PMC3731343; doi:10.1371/journal.pone.0070161)
Supplement: Text S3 — Possible grouping effects. This text investigates possible grouping effects, e.g. differences between summer districts with respect to natural and/or social factors. (PDF) [file pone.0070161.s003.pdf]

Text S3 – POSSIBLE GROUPING EFFECTS

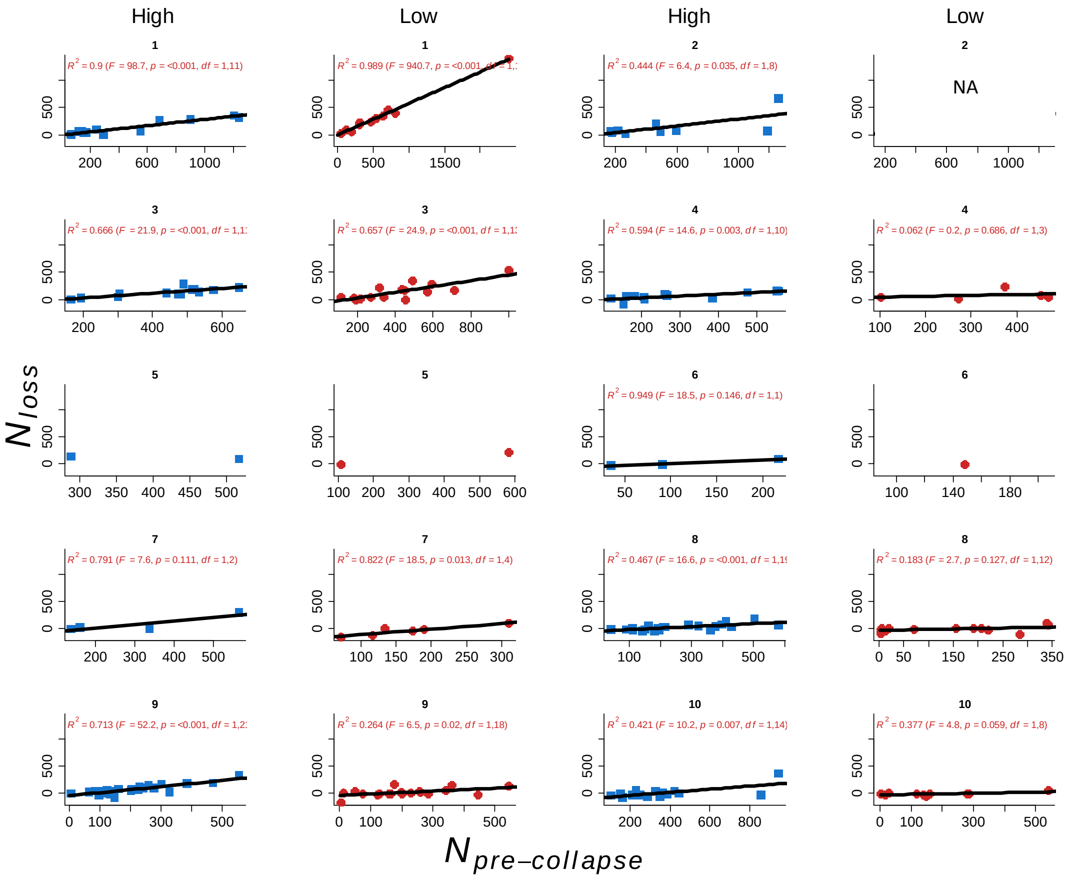

Fig. S3.1. Linear relationship between pre-collapse herd size ($N_{pre-collapse}$) and number of reindeer lost from pre-collapse to collapse (N_{loss}) for all districts in the study. The plot is arranged according to the study design described in S1 (‘High’ and ‘Low’ refers to density of reindeer while number designation refer to district pairs). The lack of fitted regression lines for a few district pairs was due to a low sample size (i.e. districts with ≤2 husbandry units).

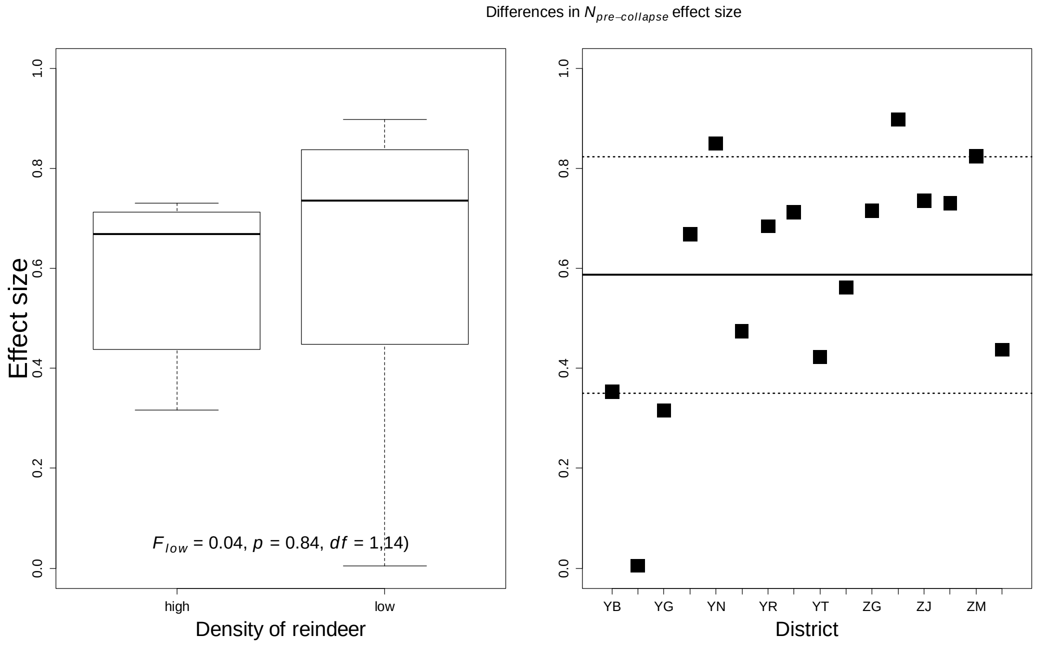

Fig. S3.2. Showing the differences in slope point estimates [i.e. the effect of pre-collapse herd size ($N_{pre-collapse}$) on the number of reindeer lost from pre-collapse to collapse (N_{loss})] from the linear models fitted in Fig. S3.1 grouped according to the study design 'High' and 'Low' density of reindeer (*left panel*) and district (*right panel*). Solid and dotted lines indicate mean and ± 1 SD for the point estimate respectively (*right panel*).

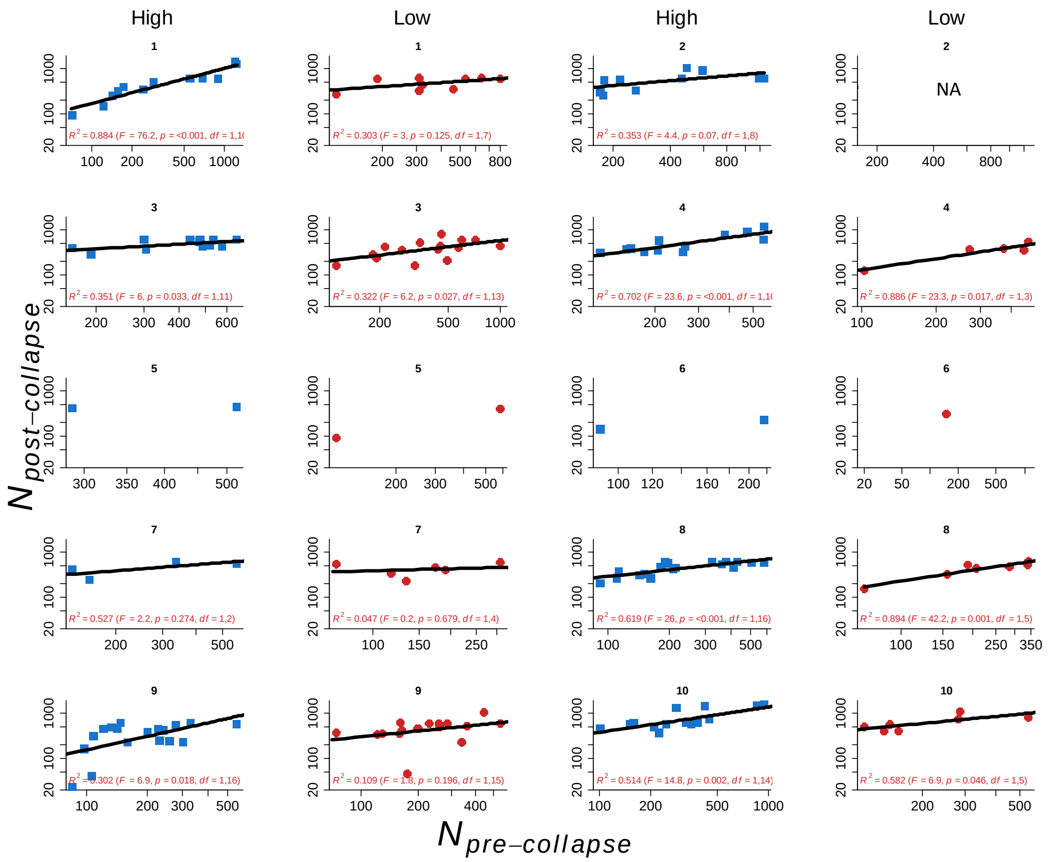

Fig. S3.3. Linear relationship (on $\log_e$-scale) between pre-collapse ($N_{pre-collapse}$) and post-collapse ($N_{post-collapse}$) herd size for all summer districts in the study. The plot is arranged according to the study design described in S1 (see also Fig. S3.1 text). The lack of fitted regression lines for a few district pairs was due to too a limited sample size (i.e. the districts with ≤2 husbandry units).

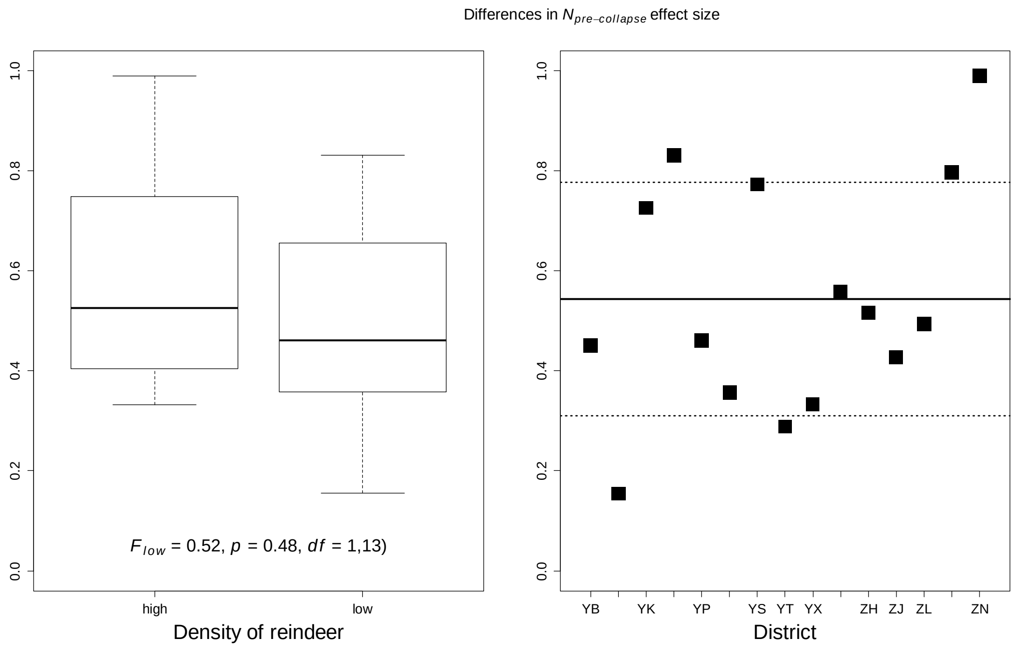

Fig. S3.4. Showing the differences in slope point estimates [i.e. the effect of pre-collapse ($N_{pre-collapse}$) on post-collapse ($N_{post-collapse}$) herd size] from the linear models fitted in Fig. S3.3 grouped according to the study design 'High' and 'Low' density of reindeer (*left panel*) and district (*right panel*). Solid and dotted lines indicate mean and ± 1 SD in point estimate respectively (*right panel*).
