# Supplementary material for: Why Herd Size Matters – Mitigating the Effects of Livestock Crashes
Source: PLoS One. 2013 Aug 1;8(8):e70161. doi: 10.1371/journal.pone.0070161 (PMC3731343; doi:10.1371/journal.pone.0070161)
Supplement: Text S4 — Finding the correct variance structure. This text explores models with different variance structures in order to assess if violations of the homoscedastic assumption altered the conclusions presented in the main text. (PDF) [file pone.0070161.s004.pdf]

Text S4 – FINDING THE CORRECT VARIANCE STRUCTURE

INTRODUCTION

Looking at the relationship between $N_{pre-collapse}$ and both (1) N_{loss} and (2) $N_{post-collapse}$ indicated problems related to heterogeneity as the variance seemed to increase for higher values of $N_{pre-collapse}$. As a consequence, we re-fitted the models using generalized least squares (GLS) with different variance structures using the function *gls* from the *nlme* package in R ([1], see [2] for a practical introduction). As this did not change the interpretation of the relationships we presented the results from regular linear models in the main text and provide the details pertaining to the GLS analyzes below

Loss

Model selection

Table S4.1. Showing the AIC values for models (N_{loss} as a function of $N_{pre-collapse}$) with different variance structures to account for heterogeneity (i.e. variance increased for higher values of $N_{pre-collapse}$). To reduce numerical instabilities due to large values in the variance covariate ($N_{pre-collapse}$), the variance covariate was rescaled to $N_{pre-collapse} / \max(N_{pre-collapse})$ (as suggested by [2]) but the un-scaled covariate was used as the fixed part of the model. The winning model models the structure of the residuals as σ^2 multiplied by an exponential function of the variance covariate $N_{pre-collapse}$ and an unknown parameter δ (see [2], p. 71-100 for details).

#	Model type	df	AIC	ΔAIC
1	Normal	3	2373.5	90.2
2	Fixed variance	3	2439.9	156.6
3	Power of the variance covariate	4	2326.3	43.0
**4**	**Exponential of the variance covariate**	**4**	**2283.3**	**0.0**

Results

Table S4.2. Estimates from GLS model relating number of reindeer lost from pre-collapse to collapse (N_{loss}) as a function of pre-collapse herd size ($N_{pre-collapse}$), fitted with an exponential variance structure (i.e. exponent of $N_{pre-collapse}$, see Table S4.1 for details).

Parameter	Response: N_{loss}		
	Value	(95% CI)	P
Intercept	-38.877	(-52.839, -24.915)	< 0.001
$N_{pre-collapse}$	0.353	(0.297, 0.409)	< 0.001
Exponent (δ)	3.512		
Residual SE:	43.003		
df_{total}	202		
$df_{residual}$	200		

Residual plots

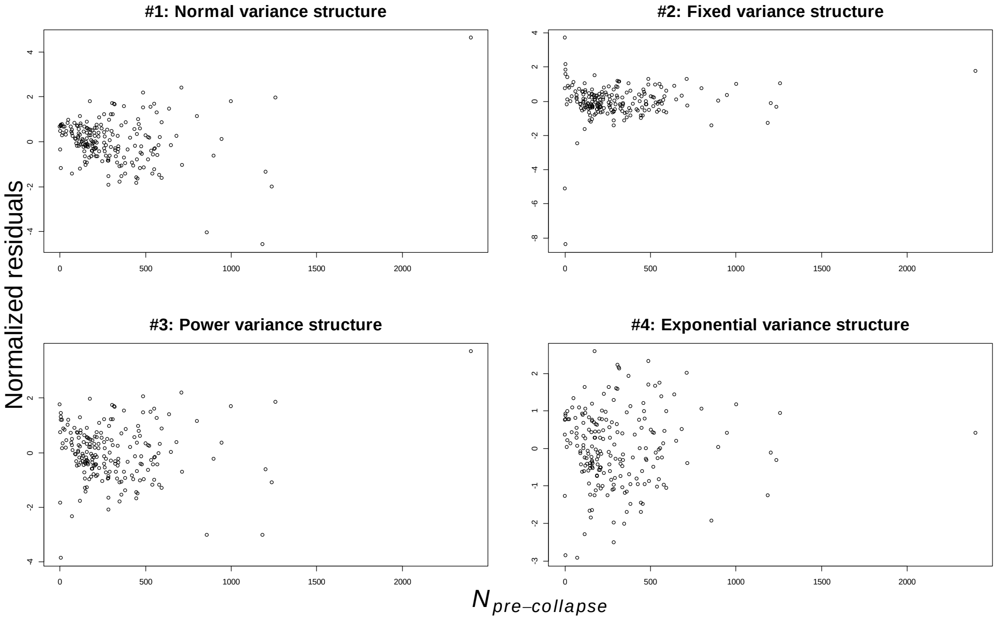

Fig. S4.1. Residual plots for the models in Table S4.1.

Post-collapse herd size

Model selection

Table S4.3. Showing the AIC values for models ($N_{post-collapse}$ as a function of $N_{pre-collapse}$) with different variance structures to account for heterogeneity (i.e. variance increased for higher values of $N_{pre-collapse}$). Due to log transformation of $N_{pre-collapse}$, no rescaling of the variance covariate was necessary. The winning model models the structure of the residuals as σ^2 multiplied with the power of the absolute value of the variance covariate $N_{pre-collapse}$ and an unknown parameter δ (which is estimated, if it equals 0, we have the linear model, see [2], p. 71-100 for details).

#	Model type	df	AIC	ΔAIC
1	Normal	3	349.9	69.5
2	Fixed variance	3	457.7	177.2
**3**	**Power of the variance covariate**	**4**	**280.5**	**0.0**
4	Exponential of the variance covariate	4	284.7	4.2

Results

Table S4.4. Estimates from GLS model relating post-collapse herd size ($N_{post-collapse}$) as a function of pre-collapse herd size ($N_{pre-collapse}$), fitted with a power variance structure (i.e. power of $N_{pre-collapse}$, see Table S4.3 for details). Note: both the response and predictor was transformed using the natural logarithm.

Parameter	Response: $N_{post-collapse}$		
	Value	(95% CI)	*P*
Intercept	2.756	(2.228, 3.284)	< 0.001
$N_{pre-collapse}$	0.587	(0.496, 0.677)	< 0.001
Power (δ)	-1.460		
Residual SE:	5.631		
df_{total}	189		
$df_{residual}$	187		

Residual plots

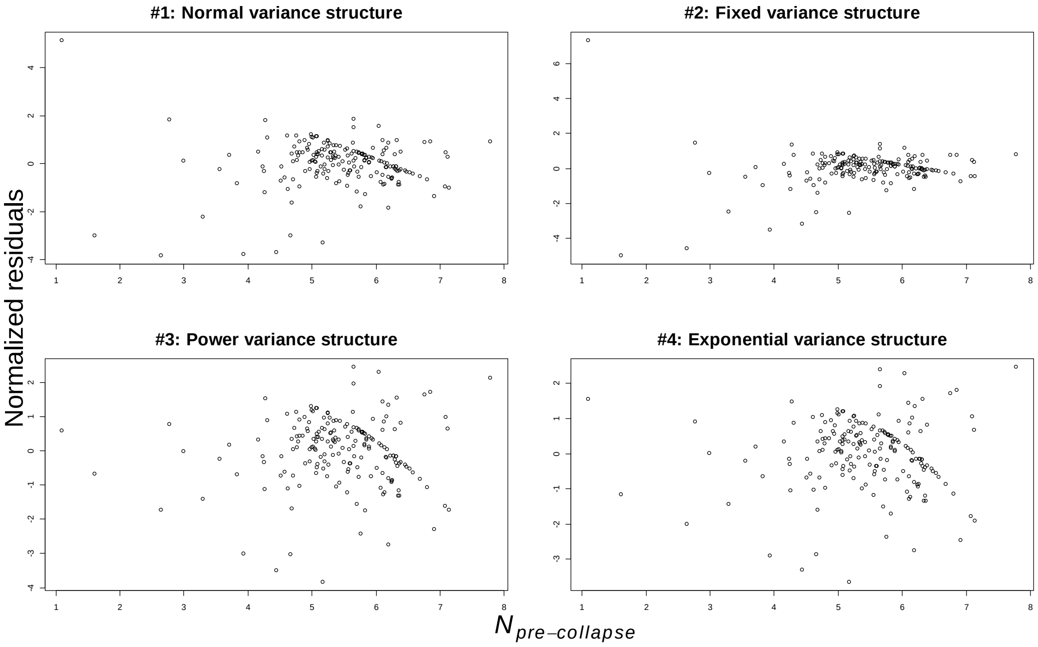

Fig. S4.2. Residual plots for the models in Table S4.3.

REFERENCES CITED

1. Pinheiro JC, Bates DM, DebRoy S, Deepayan S (2007) nlme: Linear and Nonlinear Mixed Effects Models. R package version 3.1-83.
2. Zuur AF, Ieno EN, Walker N, Saveliev AA, Smith GM. (2009). *Mixed effects models and extensions in ecology with R*, New York: Springer.
