# Supplementary material for: Why Herd Size Matters – Mitigating the Effects of Livestock Crashes
Source: PLoS One. 2013 Aug 1;8(8):e70161. doi: 10.1371/journal.pone.0070161 (PMC3731343; doi:10.1371/journal.pone.0070161)
Supplement: Text S5 — Reported loss. This text replicates the analysis pertaining to loss presented in the main text but where ‘reported loss’ is used as a response. (PDF) [file pone.0070161.s005.pdf]

Text S5 – REPORTED LOSS

ANALYSIS

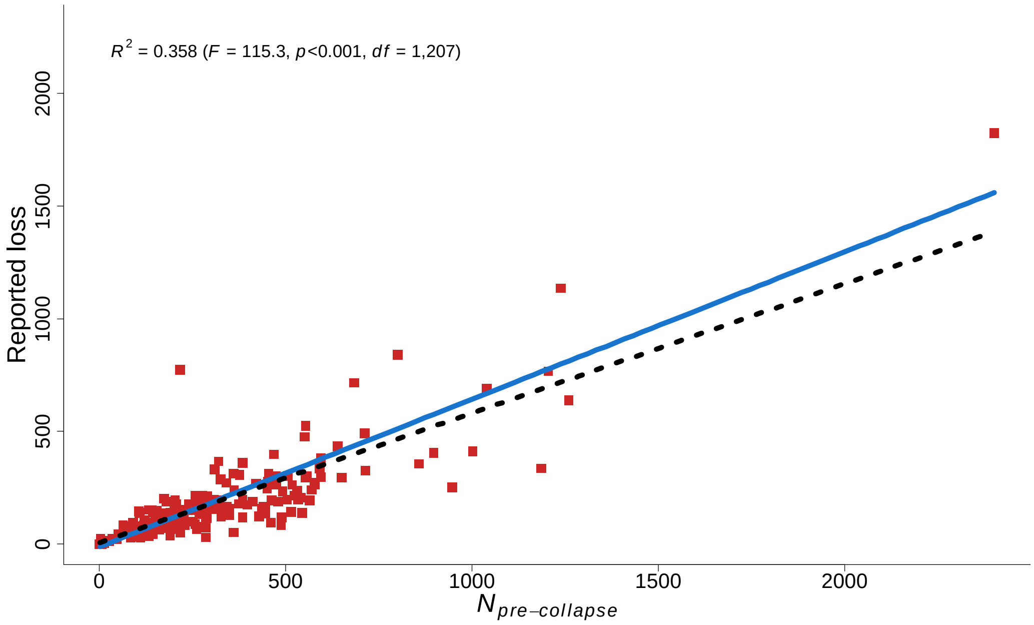

Fig S5.1. Showing the linear relationship between herd size pre-collapse ($N_{pre-collapse}$) and number of animals *reported* lost from pre-collapse to collapse ($N_{reported-loss}$). Model parameters: Intercept = 193.51 (95% CI: 160.14, 226.89) and slope ($N_{pre-collapse}$) = 0.65 (95% CI: 0.53, 0.77). The positive relationship indicates that as herd size increases, reported losses also increases, i.e. increasing herd size by one in 1998 increases expected reported losses by 0.65 reindeer. By using reported loss as a response, herd accumulation seems to be even more expensive. Note, however, that reported loss may not be accurate since losses caused by endangered predator are compensated by the Norwegian Government. This compensation is received after the damage has occurred which presents an incentive to over-report damages to gain additional income, i.e. reported losses may reflect strategic behavior rather than actual losses (cf. [1] for details). Please note that the model parameters are from fitting a model when centering $N_{pre-collapse}$ while the plot shows the non-transformed relationship. Hatched line show the relationship from a Generalized Least squares (GLS) model accounting for potential variance heterogeneity (see below for details).

FINDING THE CORRECT VARIANCE STRUCTURE

Model selection

Table S5.1. Showing the AIC values for models ($N_{reported-loss}$ as a function of $N_{pre-collapse}$) with different variance structures to account for heterogeneity (i.e. variance increased for higher values of $N_{pre-collapse}$). To reduce numerical instabilities due to large values in the variance covariate ($N_{pre-collapse}$), the variance covariate was rescaled to $N_{pre-collapse} / \max(N_{pre-collapse})$ (as suggested by [2]), but the un-scaled covariate was used as the fixed part of the model. The winning model models the structure of the residuals as σ^2 multiplied by an exponential function of the variance covariate $N_{pre-collapse}$ and an unknown parameter δ (see [2], p., 71-100 for details).

#	Model type	df	AIC	ΔAIC
1	Normal	3	2892.5	308.0
2	Fixed variance	3	2700.0	115.6
3	Power of the variance covariate	4	2634.7	50.2
**4**	**Exponential of the variance covariate**	**4**	**2584.4**	**0.0**

Results

Table S5.2. Estimates from GLS model relating number of reindeer reported lost from pre-collapse to collapse ($N_{reported-loss}$) as a function of pre-collapse herd size ($N_{pre-collapse}$), fitted with a exponential variance structure (i.e. exponent of $N_{pre-collapse}$, see Table S5.1 for details).

Parameter	Response: $N_{reported-loss}$		
	Value	(95% CI)	P
Intercept	6.428	(-5.738, 18.594)	0.302
$N_{pre-collapse}$	0.574	(0.488, 0.661)	< 0.001
Exponent (δ)	11.712		
Residual SE:	24.898		
df_{total}	209		
$df_{residual}$	207		

Residual plot

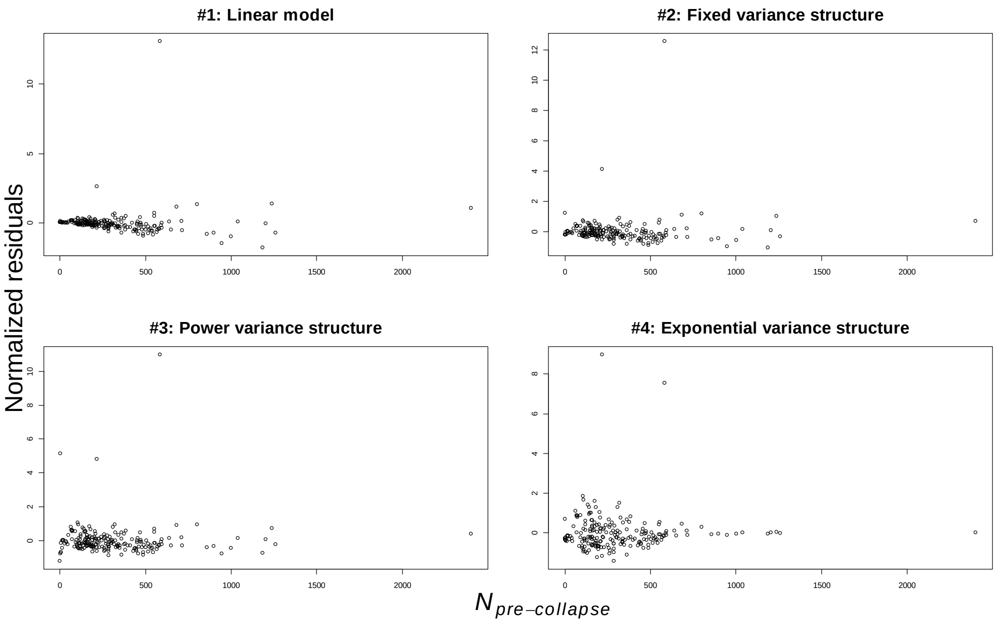

Fig. S5.2. Residual plots for the models in Table S5.1. Please note that all plots seem to indicate the presence of extreme values. While the removal of 2 observations improved the residual plots, it had no effect on the results from the model selection. Also, while the removal reduced the effect size of $N_{pre-collapse}$ by 12.7% [the point estimate changed from 0.574 (95% CI: 0.488, 0.661) to 0.501 (95% CI: 0.4451, 0.551)] we kept the observations in the analyses as the removal did not change the direction of the point estimate and thus had no impact on the inferences drawn from the analysis.

REFERENCES CITED

1. Næss MW, Bårdsen B-J, Pedersen E, Tveraa T (2011) Pastoral herding strategies and governmental management objectives: predation compensation as a risk buffering strategy in the Saami reindeer husbandry. Human Ecology 39: 489-508.
2. Zuur AF, Ieno EN, Walker N, Saveliev AA, Smith GM. (2009). *Mixed effects models and extensions in ecology with R*, New York: Springer.
